# Piano playing-associated musculoskeletal symptoms among tertiary education students in China: the role of playing postures, habits, and emotional states

**DOI:** 10.3389/fpsyg.2025.1601818

**Published:** 2025-09-10

**Authors:** Xueying Zhang, Dawen Li

**Affiliations:** School of Arts, Sun Yat-sen University, Guangzhou, China

**Keywords:** China, emotions, piano, playing habits, playing postures, playing-associated musculoskeletal symptoms

## Abstract

**Background:**

Given that research on the prevalence of musculoskeletal symptoms and their associated factors among Chinese piano players remains scarce, this study, situated within the local Chinese cultural context, investigates the current status of playing-associated musculoskeletal symptoms (PAMSS) among piano-playing students in tertiary institutions in China.

**Objective:**

This study examines the prevalence and characteristics of PAMSS among piano-playing students in Chinese universities, focusing on two contributing factors: playing postures and playing habits. It further explores the relationships between these symptoms and multidimensional emotional states.

**Methods:**

A cross-sectional design was employed. The Chinese Musculoskeletal Questionnaire (CMQ) and the Chinese version of the Positive and Negative Affect Schedule–Expanded (PANAS-X) were administered to 106 university piano learners, including both professional and amateur students. Descriptive statistics, between-group comparisons, correlation analyses, and binary logistic regression models were used to: (a) examine the relationships between PAMSS, playing postures, and playing habits; and (b) explore associations between symptoms and emotional states.

**Results:**

Among Chinese college piano students, the most frequently reported PAMSS occurred in the neck, shoulders, wrists/hands, and back, primarily affecting the upper limbs, with an overall prevalence of approximately 50%. Playing postures (e.g., seat height settings, wrist postures, shoulder abduction) and playing habits (e.g., relaxation after key touch, breathing patterns) were significantly associated with PAMSS. Symptoms were also associated with three negative emotions—hostility, guilt, and sadness—and with surprise, but no significant association was observed with positive emotions. Female players were more likely to report PAMSS than male players, although this difference warrants further investigation to identify potential contributing factors such as anatomical differences or practice intensity.

**Conclusion:**

To reduce the occurrence of PAMSS, piano students should adopt proper playing postures, including maintaining a near-neutral wrist position, keeping the shoulders naturally relaxed and dropped, and avoiding excessive shoulder shrugging. Conscious, regular breathing and prompt relaxation after key touch may also help prevent musculoskeletal problems. While this study identified correlations between PAMSS and emotional states, the bidirectional nature of these relationships requires longitudinal research to determine possible causal mechanisms. Limitations include reliance on self-reported data and restricted sample generalizability, both of which require further validation in future studies.

## 1 Introduction

For Since ancient Greece, people have admired athletes not only as outstanding representations of human strength and speed, but also for their perseverance and determination in overcoming physical and mental obstacles. However, no matter how advanced technology becomes, preventing and treating athletes’ injuries and illnesses remains a difficult problem that urgently needs to be addressed. The field of music performance faces the same challenges (Bird and Macdonald, 2013; Stanhope, 2016), especially for the numerous piano players. As piano players often face a large audience alone, they experience high mental stress. At the same time, piano playing demands exceptional skill and precision, requiring the coordination and high-frequency movement of multiple body parts and joints. Its complexity and intensity are comparable to those of competitive sports. Therefore, the physical and mental challenges faced by pianists are not only universal but also highly representative.

### 1.1 Playing-associated musculoskeletal symptoms (PAMSS)

Musculoskeletal disorders (MSDs) refer to chronic strain-related pathological changes in various parts of the body, such as the shoulders, neck, and lower back. These disorders can cause pain, discomfort, and functional impairments, leading to a decline in the work capacity of the occupational population (Ackermann et al., 2012; Wang et al., 2017). This definition has been adopted in the commonly used Standardized Nordic Musculoskeletal Questionnaire and has undergone extensive validation (Kuorinka et al., 1987; Arsalani et al., 2011; López-Aragón et al., 2017).

In the field of music performance, Zaza et al. introduced playing-related musculoskeletal disorders (PRMD) as a specific designation for MSDs in musicians, requiring that “symptoms affect the normal level of performance” and explicitly excluding mild discomfort (Zaza et al., 1998). However, subsequent studies revealed large variations in PRMD prevalence depending on whether mild symptoms were included—ranging from 29 to 90% (Rousseau et al., 2023; Li et al., 2024). Notably, among university students, while 62% reported symptoms, only 28% met PRMD criteria (Li et al., 2024).

Therefore, this study adopts the concept of *playing-associated musculoskeletal symptoms* (PAMSS) to capture early-stage or subclinical symptoms among Chinese piano students (e.g., persistent discomfort not yet affecting performance ability) (Stanhope and Milanese, 2016; Stanhope et al., 2019). The term *“associated”* is intentionally used instead of *“related”* to emphasize a statistical association between the symptoms and the three factors measured in this study. Given the multifactorial nature of musculoskeletal symptom development, no causal assumptions are made. Moreover, the use of PAMSS aligns with the measurement definition and framework of the Chinese Musculoskeletal Questionnaire (CMQ), which defines symptoms lasting ≥24 h and does not require functional impairment. Additionally, PAMSS differs from PRMD in excluding the performance-impairment threshold, ensuring strict consistency between the symptom definition (persistent symptoms) and the methodological framework (CMQ). This approach complies with the Musculoskeletal Health Surveillance Guidelines for Performing Artists (International Association for Dance Medicine and Science International Federation of Musculoskeletal Medicine for Performing Artists, 2022), while also preventing conceptual confusion with Zaza’s original definition.

Recent research suggests that the development of PAMSS can be effectively explained through the biopsychosocial model (Gatchel et al., 2007). This model highlights three interrelated dimensions: biomechanical factors (e.g., sustained shoulder abduction postures) that directly contribute to tissue microtrauma (Ackermann et al., 2012; Ballenberger et al., 2023); psychological factors (e.g., performance anxiety, negative emotions) that exacerbate symptoms by increasing neuromuscular tension (Cruder et al., 2023; Stanhope et al., 2022); and sociobehavioral factors (e.g., training intensity, lack of preventive knowledge) that foster symptom chronicity (Miao et al., 2024).

Building on this framework, Guptill emphasized the lived experiences of professional musicians, showing how playing-associated injuries can lead to identity disruption, emotional distress, and social role conflicts (Guptill, 2008). Her work extended the biopsychosocial model by enhancing its explanatory power within the context of music performance. This model provides the theoretical foundation for the present study’s exploration of the multidimensional associations among playing postures, habits, and emotional states.

It is noteworthy that this multifactorial interactions mechanism. The interaction of these factors may amplify symptom severity and have long-term consequences for performers. Numerous studies demonstrate that untreated PAMSS can progress to functional impairments, resulting in diminished performance quality, interrupted practice, career termination, and lifelong negative impacts on physical and mental health (Ackermann et al., 2012; Silva et al., 2015; Stanhope et al., 2022). More alarmingly, most piano students lack systematic awareness of musculoskeletal health—they neither receive preventive training nor recognize early warning signs until symptoms escalate into irreversible injuries (Spahn et al., 2002; Ackermann et al., 2012; Zhao et al., 2024). This cognitive gap highlights the necessity and urgency of early-stage research based on the biopsychosocial model.

### 1.2 Playing posture and playing habits

Playing postures and playing habits are two significant associated factors for PAMSS. Improper playing postures and habits can cause skeletal muscle fatigue and soreness. In more severe cases, they can lead to pain, strain, and lifelong consequences (Spahn et al., 2002; Kok et al., 2016; Rousseau et al., 2023). Quantitative research shows that incorrect sitting postures (Blanco-Piñeiro et al., 2015; Araújo et al., 2017; Li et al., 2024), inappropriate seat height (Li et al., 2024), improper wrist postures (Allsop and Ackland, 2010; Dommerholt, 2010; Kok et al., 2016; Turner et al., 2023; Zhao et al., 2024), shoulder postures (Spahn et al., 2002; Allsop and Ackland, 2010; Kok et al., 2016; Li et al., 2024), and elbow postures (Spahn et al., 2002; Li et al., 2024) in piano performance can influence PAMSS.

International studies illustrate this clearly: Allsop and Ackland found that incorrect wrist and shoulder postures influence PAMSS (Allsop and Ackland, 2010); Ackermann and Kenny found that unnatural shoulder and neck postures were associated with PAMSS (Ackermann et al., 2012); Blanco-Piñeiro et al. indicated that incorrect shoulder abduction and sitting postures affect PAMSS (Blanco-Piñeiro et al., 2015); and Kok et al. noted that incorrect shoulder positions (including abduction and shrugging) and wrist postures can affect musicians’ musculoskeletal health (Kok et al., 2016).

In terms of playing habits, research increasingly focuses on breathing awareness, breathing states, and relaxation after key touch. For example, Mark emphasized that conscious breathing helps prevent unnecessary muscle tension (Mark et al., 2003). Furuya et al. noted that excessive muscle tension in performers increases the likelihood of developing PAMSS, with failure to relax after key touch being one manifestation (Furuya et al., 2006). Recent longitudinal studies confirm the importance of playing habits: Ballenberger et al. found significant associations between specific playing postures/habits and musculoskeletal symptoms in music students (Ballenberger et al., 2023); Cruder et al. reported that changes in playing habits increased PAMSS risk (Cruder et al., 2023); Zhao et al. found that relaxation awareness and warm-up habits influence PAMSS (Zhao et al., 2024). Stanhope also suggested that subjective physiological states, such as heightened performance awareness, may influence symptom onset and progression (Stanhope et al., 2022).

However, the subjects of the above-mentioned studies were all foreign pianists, so these findings may not be directly applicable to Chinese piano students. Although several domestic quantitative studies have investigated PAMSS among Chinese pianists, they have limitations in questionnaire validity and statistical analysis, which may affect their credibility. For example, Li et al. found that elbow and shoulder abduction and seat height are likely to cause musculoskeletal discomfort, with a reported symptom prevalence of 90% among Chinese students (Li et al., 2024). However, their measurement tool was self-developed, and they did not report test–retest reliability or conduct confirmatory factor analysis. Similarly, Zhao et al. found that incorrect wrist and sitting postures are likely to cause muscle fatigue (Zhao et al., 2024), but their questionnaire was developed independently by a Malaysian higher-education institution, with incomplete reliability/validity testing and no cross-cultural adaptation.

There are also disagreements in the literature about the impact of certain postural variables. For example, Furuya and Zhao believe that elbow joint posture is associated with musculoskeletal symptoms (Furuya et al., 2006; Zhao et al., 2024), while Allsop, Ackland, and Li argue that it has no impact (Allsop and Ackland, 2010; Li et al., 2024). Such inconsistencies may reflect differences in study design, operational definitions, or individual variability. Further investigation is needed to clarify these relationships.

In addition to research articles, numerous pedagogical books address piano playing postures and habits. Mark discussed the impact of correct sitting posture and seat height, breathing in piano playing, and the importance of conscious relaxation to avoid muscle tension (Mark et al., 2003). Hamilton advised that elbows should remain relaxed and naturally lowered, and also stressed the importance of relaxation after key touch (Hamilton, 2012). Although these works are based on teaching experience and observation rather than empirical research, they offer valuable insights. Thus, it is necessary to investigate scientifically how these postures and habits influence PAMSS development.

In summary, playing postures and playing habits are associated factors for PAMSS. While international research provides preliminary evidence, validation in Chinese piano students remains limited. This study focuses on sitting posture, wrist posture, shoulder abduction posture, and elbow posture, as well as breathing awareness, breathing state, and relaxation after key touch. The goal is to determine their association with PAMSS through quantitative analysis, thereby improving understanding of the factors influencing musculoskeletal symptoms in piano playing.

The biopsychosocial model emphasizes the bidirectional relationship between psychological factors and physical symptoms (Gatchel et al., 2007; Guptill, 2008). Music Performance Anxiety (MPA) refers to anxious emotions experienced in performance situations, influenced by biological susceptibility, psychological factors, early experiences, and current performance status. MPA affects physiological responses, cognitive processes, and behavior (Kenny, 2023). Kenny found a positive correlation between the severity of musculoskeletal symptoms and MPA severity: high anxiety can lead to elevated heart rate and muscle tension, causing stiffness—a potential early sign of musculoskeletal symptoms. Persistent tension may result in chronic muscle and joint strain, increasing PAMSS risk.

### 1.3 Psychological states

Beyond anxiety, PAMSS may be linked to a range of psychological states. Kenny and Ackermann (2014) demonstrated a significant association between emotional states and physical symptoms in professional orchestral musicians. Cruder et al. (2023) noted that PAMSS may not only trigger negative emotions but also contribute to psychological problems. Stanhope et al. (2022) suggested that changes in psychological state may influence symptom development and progression. Herman and Clark (2023) proposed reconceptualizing MPA, suggesting that a positive mindset toward performance stress may help reduce PAMSS. Using transcendental phenomenology, Miao et al. (2024) found relationships between musculoskeletal symptoms and emotions such as sadness, depression, helplessness, and despair. Based on this research, we hypothesize that the psychological impact of PAMSS is not limited to MPA but extends to broader emotional dimensions. However, relevant research is scarce. Therefore, we aim to explore the relationship between PAMSS and multidimensional emotional states to broaden understanding of these connections.

This study, based on the Chinese cultural context, investigates PAMSS among piano students, focusing on modifiable factors including playing posture (e.g., incorrect sitting posture, hand position, wrist alignment, shoulder posture, elbow position) and playing habits (e.g., breathing patterns, relaxation after key touch). It also examines the impact of PAMSS on the emotional states of Chinese tertiary-level piano students.

Guided by the biopsychosocial model, this cross-sectional study seeks to determine the prevalence and characteristics of PAMSS among university-level piano performers in China, providing empirical evidence to inform culturally tailored interventions for this population.

## 2 Materials and methods

### 2.1 Participants

The research respondents were piano-playing students (including non-professional and professional students) from comprehensive universities, normal colleges and universities, and conservatories of music in different parts of China, such as Beijing, Guangdong Province, Guangxi Province, Shandong Province, and Zhejiang Province. The questionnaire recruitment period lasted 3 months. During this time, the survey was distributed and collected online via the network platforms of various universities and music departments.

### 2.2 Selection criteria

Inclusion criteria required participants to be registered piano students currently studying at higher education institutions within the relevant regions, including comprehensive universities, normal universities, and conservatories of music. A total of 204 questionnaires were distributed, and 204 were collected.

Exclusion criteria comprised incomplete questionnaire responses, data inconsistencies (such as reported years of piano study exceeding actual age), and contradictory or inconsistent answers related to pain symptoms.

**Classification Criteria for Significant PAMSS:** Participants were classified into the significant PAMSS group if they met the following criteria: (1) Reported musculoskeletal symptoms in ≥2 of the 12 body regions (neck, shoulders, upper back, elbows, wrists/hands, lower back, hips/thighs, knees, ankles/feet, forearms, upper arms, and calves). (2) Symptoms lasting more than 24 h. (3) Symptom onset or aggravation linked to piano practice.

This operational definition is consistent with the CMQ’s symptom screening criteria (Qin et al., 2018).

### 2.3 Sample size and bias

A total of 204 questionnaires were distributed, and 204 were collected. As a result, the final number of valid questionnaires was 106, with a valid rate of 51.96%. Among them, there were 28 males and 78 females. In terms of school type, there were 47 piano-major students from conservatories of music, 41 piano-major students from comprehensive universities and normal colleges, and 18 non-professional piano enthusiasts from different universities. In terms of academic years, there were 20 Year 1 undergraduates, 17 Year 2 undergraduates, 23 Year 3 undergraduates, 16 Year 4 undergraduates, 11 Year 1 postgraduates, 6 Year 2 postgraduates, and 13 Year 3 postgraduates.

However, the study did not conduct a formal power analysis, which limits the ability to assess whether the sample size was adequate. Although we implemented multi-site recruitment to mitigate regional bias, the possibility of self-selection bias remains a concern. Specifically, the online recruitment method likely attracted participants who were interested in the research topic, including those already experiencing musculoskeletal symptoms. Future research should consider employing methods such as random sampling to minimize such biases and conducting a power analysis to ensure sample size adequacy.

### 2.4 Procedures

In this study, data were collected through online questionnaires. The research obtained ethical approval from the Ethics Committee of the School of Arts, Sun Yat-sen University. At the beginning of the research, the researchers selected subjects using the simple sampling method. The subjects logged in to the Wenjuanxing website^1^ to fill out the questionnaires. Before filling out the questionnaires, the subjects were informed about the background of the study and were required to sign the informed consent form. After completing the above preparation procedures, the survey was conducted anonymously. The research team also strictly followed regulations on personal data usage to ensure the security of the subjects’ personal information.

### 2.5 Tools

#### 2.5.1 Questionnaire

The questionnaire consists of six parts, as follows:

1. **Introduction:** Introduction of research team members, research background, research objectives, an overview of the questionnaire content, confidentiality of the questionnaire, and informed consent of the respondents.
2. **Demographic information:** Basic information includes: gender, age, type of student (piano-major students in conservatories of music/piano-major students in comprehensive universities and normal colleges and universities/non-professional piano enthusiasts in universities), and academic year (undergraduate/postgraduate).
3. **Playing habits:** The questions and options regarding playing habits in the questionnaire are summarized based on existing literature and teaching books, as follows:

- Do you have the habit of warming up before piano playing/practicing? Yes/No
- Breathing awareness: Do you consciously breathe while playing the piano? Rarely notice breath/Occasionally remember to breathe when there is a rest in the piece/Maintain consistent and regular breath.
- Breathing States: Is your breathing natural and relaxed during the playing process? Feel chest tightness and have difficulty inhaling/Need chest lifting to assist with inhalation/Can breathe freely and naturally without assistance.
- After playing each note /during the relaxation state after key press, what do you do? Continue to exert force and rarely relax after key press/Occasionally relax after key press/Always relax after key press.
- **There are a total of six questions regarding playing postures, five of which use illustrated examples to assist with selecting the options:** The definitions of *“natural”* and *“non-natural”* postures used in this study are based on descriptions and criteria referenced in the literature (see below). These categorizations reflect commonly recognized ergonomic and pedagogical standards rather than absolute or universally fixed classifications. The terms are employed here as operational descriptors grounded in current research consensus.

##### 2.5.1.1 Posture classification criteria

This study adopted operational definitions of *“natural”* based on established ergonomic and piano pedagogy literature. Postural assessment focused on six joint-related variables: sitting posture, elbow flexion angle, seat height, shoulder abduction, wrist alignment, and shoulder elevation. Participants reported or were evaluated on each variable through self-report and/or observational methods. Based on the following criteria, participants’ postures were categorized as either *“natural”* or *“non-natural*”:

Natural postures:

**Sitting posture:** The torso remains upright with the center of gravity aligned vertically over the ischial tuberosities; the back is naturally straight without leaning forward or slumping (Mark et al., 2003; Milanovic, 2014; Blanco-Piñeiro et al., 2015; Wong et al., 2022).

**Elbow flexion:** The elbows should be positioned in front of the torso with a flexion angle slightly greater than 90 degrees (Lister-Sink, 2002; Milanovic, 2014; Blanco-Piñeiro et al., 2015).

**Seat height:** Adjusted to maintain approximate forearm parallelism with the keyboard, with elbows positioned 1–2 cm below key level to prevent excessive wrist deviation or compensatory shoulder elevation (Hamilton, 2012; Bernstein, 2015; Leimer and Gieseking, 2019).

**Shoulder abduction:** Shoulders should remain naturally relaxed and dropped, with the upper arms loosely hanging and not pressed tightly against the torso, helping to reduce deltoid and upper trapezius tension (Lister-Sink, 2002; Mark et al., 2003; Blanco-Piñeiro et al., 2015; Kleinman and Buckoke, 2018; Wong et al., 2022).

**Wrist posture:** The wrist should remain in a near-neutral position, avoiding noticeable upward or downward deviation, and should move within the range of the neutral plane (Mark et al., 2003; Hamilton, 2012; Milanovic, 2014; Wong et al., 2022; Takamizawa and Kenway, 2023).

**Shoulder shrugging condition:** Shoulder lifting or elevation should be avoided during performance to minimize unnecessary muscle tension in the neck and shoulder area (Mark et al., 2003; Wong et al., 2022).

Postures deviating from any of the above criteria were categorized as non-natural. This classification system was developed to enhance inter-rater reliability in postural assessment and to minimize subjective interpretation in data analysis.

- Sitting Postures

**Figure 1:**
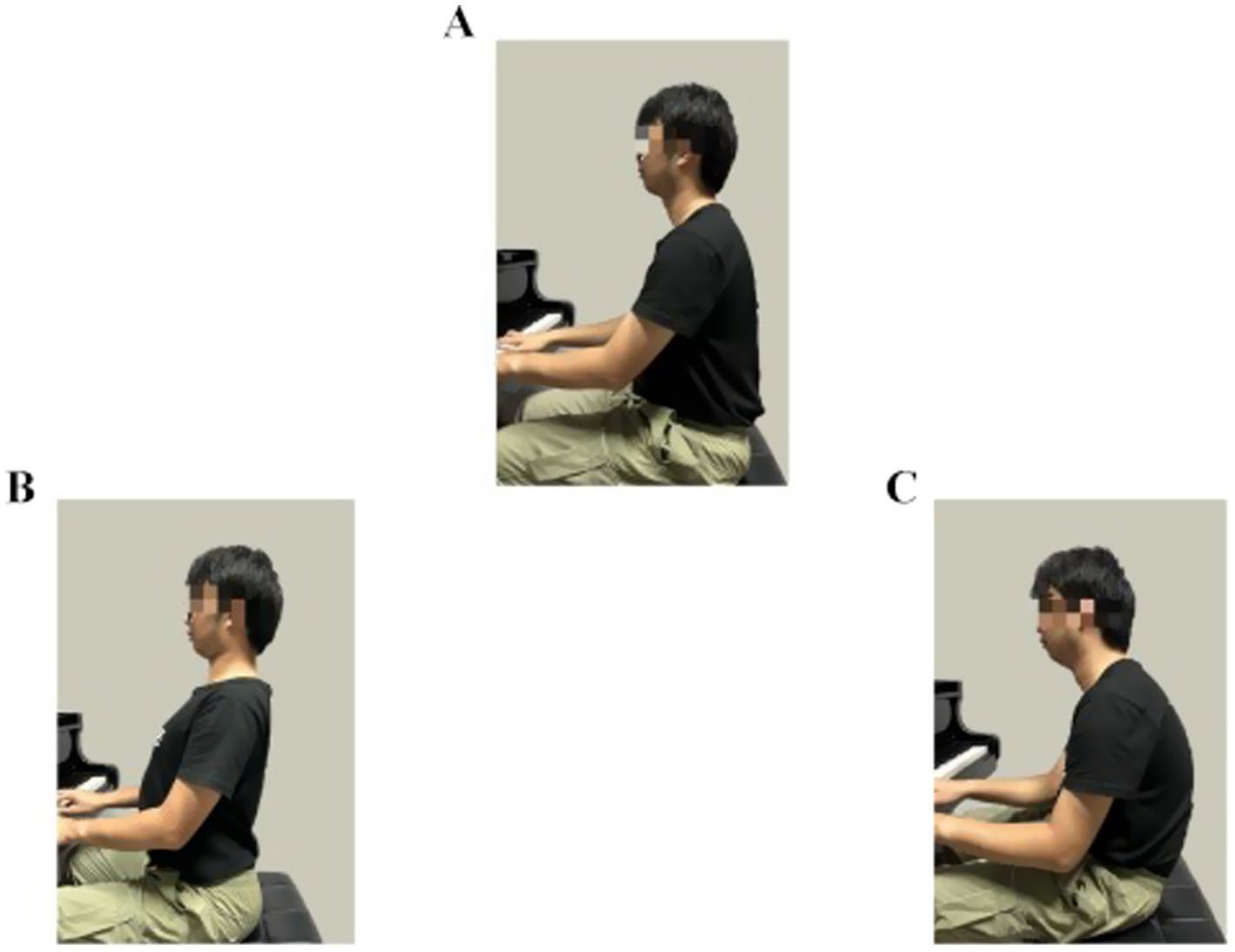
**(A)** Mild kyphotic sitting, **(B)** natural vertical sitting, **(C)** mild lordotic sitting.

Please select your sitting posture based on the images (see Figure 1).

- Elbow Flexion

**Figure 2:**
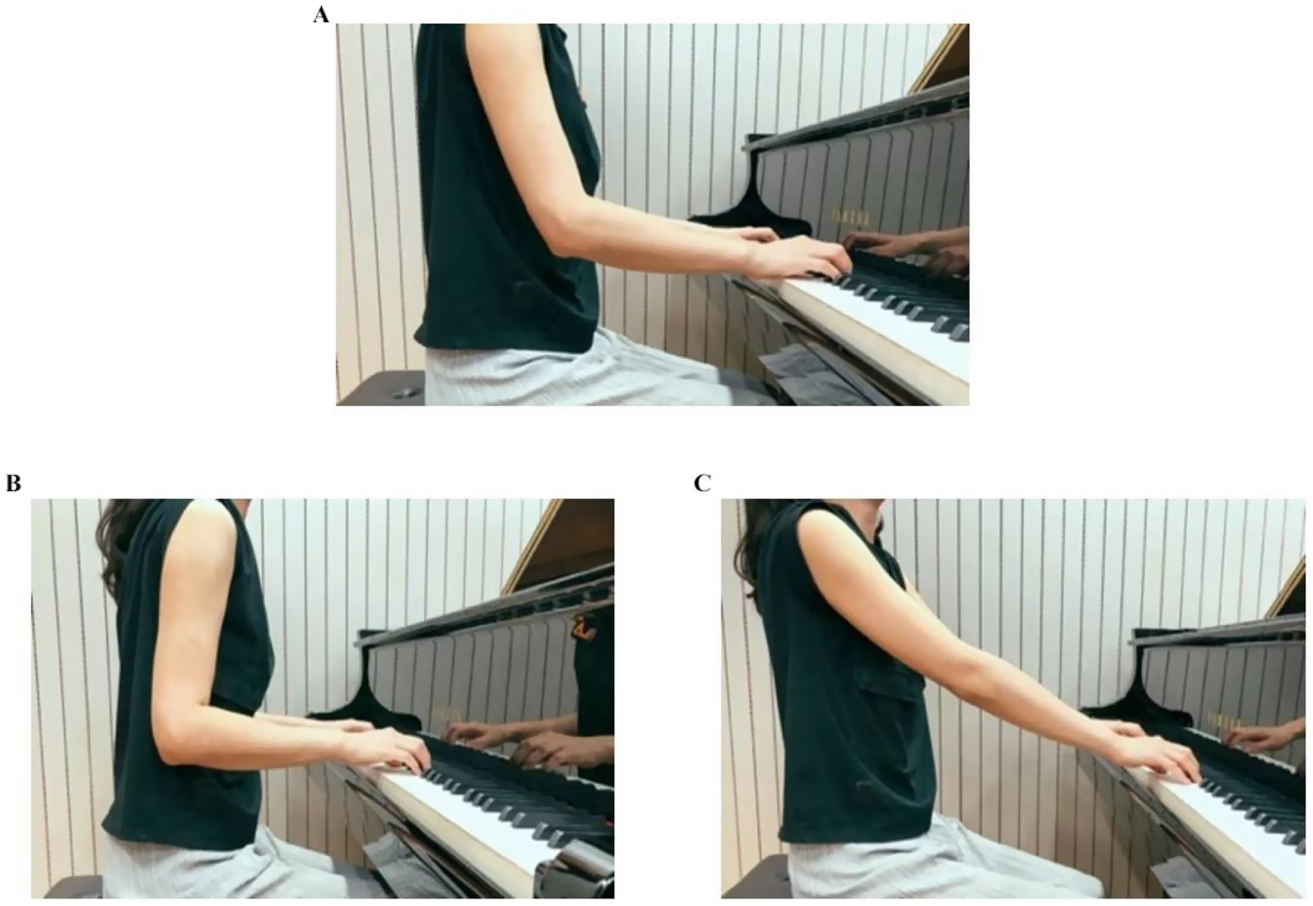
**(A)** Elbow joint angle > 90° slightly, **(B)** Elbow joint angle < 90° slightly, **(C)** Elbow joint angle > 120° slightly.

Please select your elbow flexion based on the images (see Figure 2).

- Chair Height Settings

**Figure 3:**
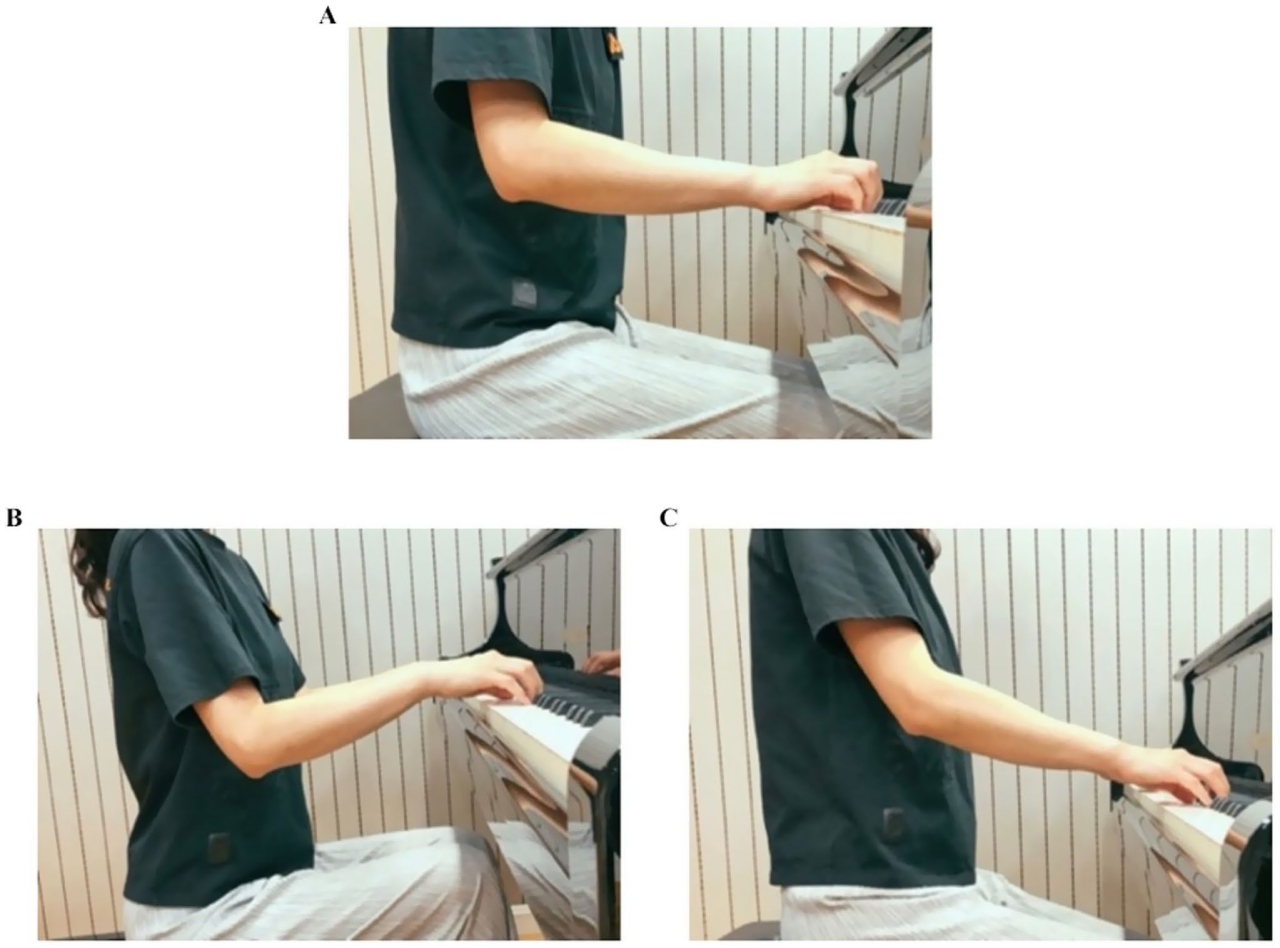
**(A)** Elbow joint at the same level as keyboard level (Natural Posture), **(B)** Elbow joint below the keyboard level, **(C)** Elbow joint above the keyboard level.

Please select your chair height setting based on the images (see Figure 3).

- Shoulder Abduction Postures

**Figure 4:**
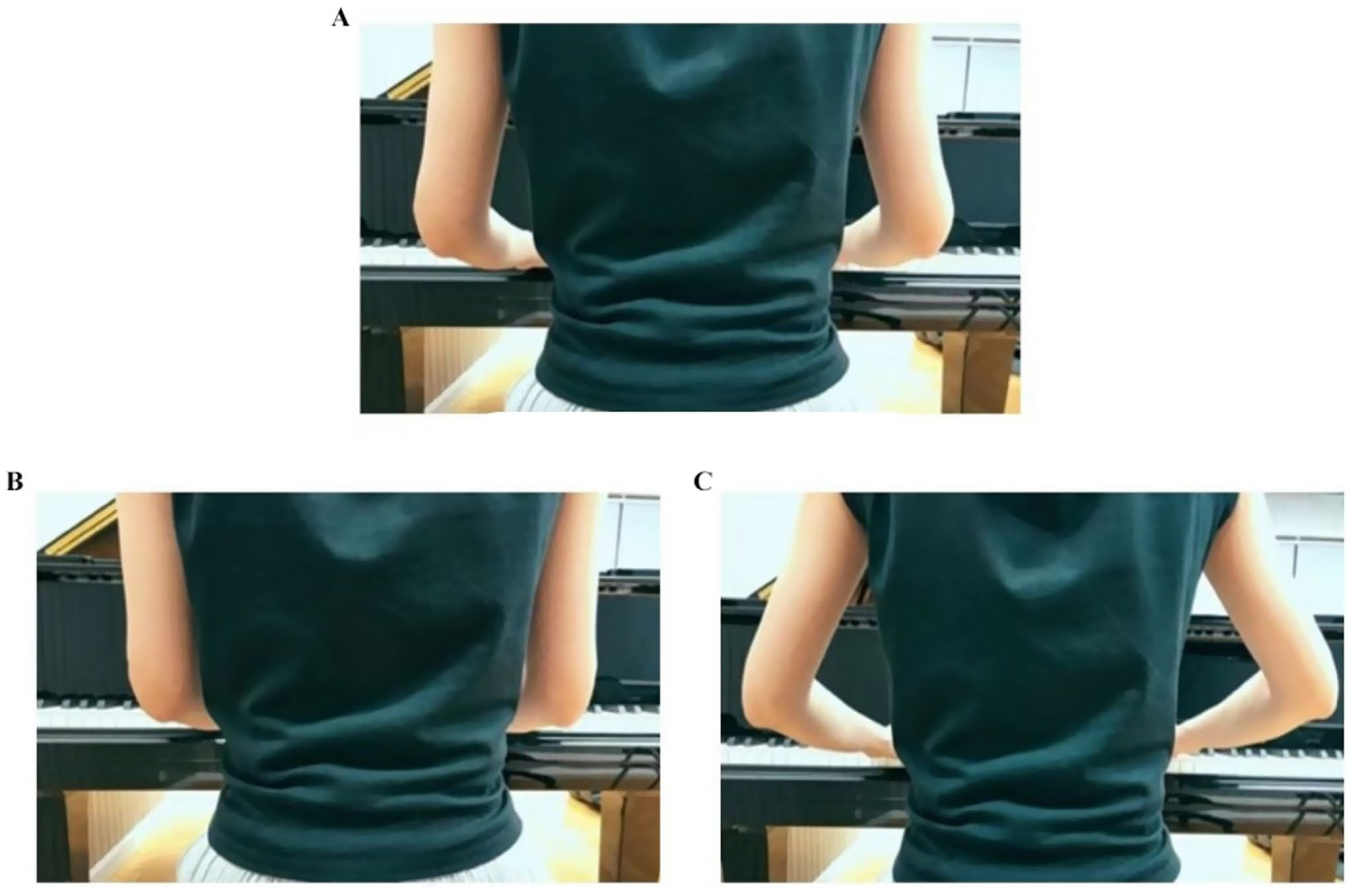
**(A)** Natural drooping (width equal to shoulders), **(B)** slightly clamped inwards, **(C)** slightly abducted outwards.

Please select your shoulder abduction posture based on the images below (see Figure 4).

- Wrist Postures

**Figure 5:**
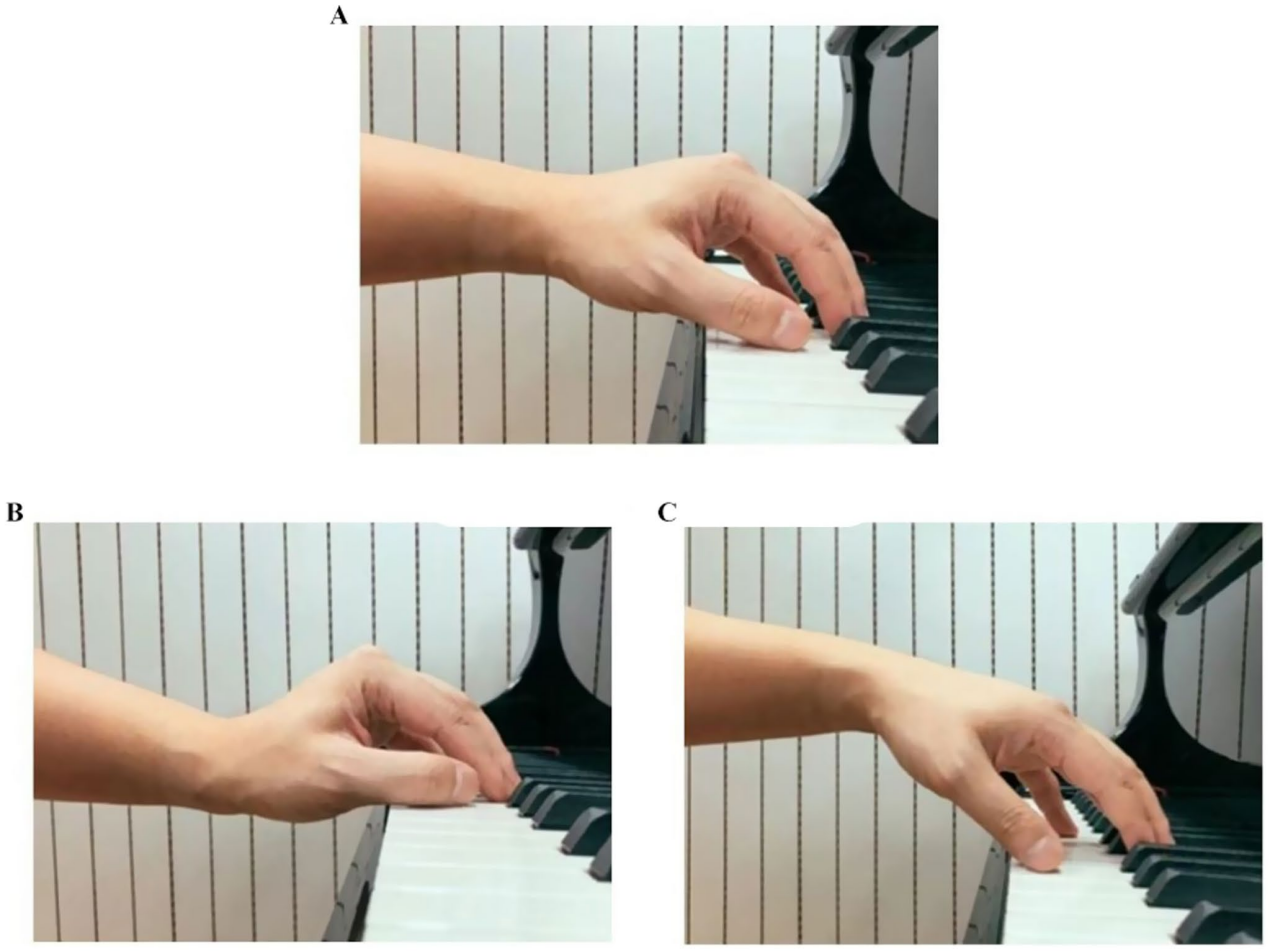
**(A)** Wrist in near-neutral position, **(B)** wrist in slightly flexed with downward deviation, **(C)** wrist in slightly extended with upward deviation.

Please select your wrist postures based on the images below (see Figure 5).

- Shoulder Shrugging Condition

Shoulder shrugging is recognized as an incorrect posture in piano performance. This study defined three shoulder shrugging conditions during piano playing: always shrugging/occasionally shrugging/never shrugging.

#### 2.5.2 Chinese Musculoskeletal Questionnaire (CMQ)

The research team, consisting of Dong Yidan, Nazhakaiti Maimaiti, Wang Fujian, Jin Xu, Wang Sheng, He Lihua, Yu Shanfa, Zhang Zhongbin, Wang Ying, Sheng Ligang, etc., developed the CMQ, which is applicable to the study of musculoskeletal disorders in the Chinese population (Qin et al., 2018). Initially, it was used for the investigation of musculoskeletal disorders among manufacturing workers. Subsequently, it has been applied and validated in different industries and occupational groups (Qin et al., 2018; Dong et al., 2020).

The questionnaire consists of 48 items and is divided into five parts: general situations, job status, health condition, social-psychological wellbeing, job environment, and job system. It adopts the 5-point Likert scale format and is accompanied by schematic diagrams of relevant body parts, which facilitate understanding and answering. The item reliability of the CMQ ranges from 0.205 to 0.841, the composite reliability from 0.545 to 0.894, and the convergence efficiency from 0.377 to 0.834, ensuring good reliability and validity (Qin et al., 2018; Dong et al., 2020). As the schematic diagrams of body parts used are consistent with those of the Standardized Nordic Musculoskeletal Questionnaire (NMQ) (López-Aragón et al., 2017), the CMQ has added new items, such as a 0–10 rating of pain or discomfort in each part, enabling a more comprehensive investigation of musculoskeletal symptoms.

The CMQ was selected for this study based on two rigorously defined criteria:

**Alignment with study objectives:** The CMQ is well-suited for detecting early-stage musculoskeletal symptoms among pianists, including mild discomfort that has not yet impaired performance ability. Unlike professional musicians, university-level student performers may experience early-stage symptoms that remain subclinical, making it essential to use tools sensitive to these subtler manifestations. It defines symptoms as those persisting for ≥24 h without requiring functional limitation. This characteristic is also the reason for not selecting other tools, such as the MPIIQM, which require functional impairment to assess the subject’s condition. Moreover, the CMQ directly supports the operational definition of PAMSS used in this study.

**Cultural validity:** The CMQ was developed within the context of Chinese occupational health, featuring language and content culturally adapted to Chinese populations. Its contextual relevance extends specifically to Chinese conservatory training environments. Compared to tools like the NMQ, it provides better linguistic and contextual appropriateness for Chinese respondents.

However, some items in the CMQ, such as those related to job status, social-psychological wellbeing, and job environment and systems, are tailored to the working environment of manufacturing groups and do not match the situation of piano playing. Therefore, while ensuring that the overall structure of the questionnaire remained unchanged, this study only selected the items related to health conditions in the CMQ and revised them according to the actual situation, so that it could be used solely as an investigation and assessment tool for musculoskeletal disorders.

The revised content includes adding potentially affected parts (such as forearms, upper arms, calves) to the schematic diagrams of body parts and excluding the history of musculoskeletal disorders or trauma unrelated to piano playing.

#### 2.5.3 Positive and negative affect schedule-expanded (PANAS-X, Chinese version)

The original Positive and Negative Affect Schedule–Expanded (PANAS-X) was developed by Watson et al. (1988). It can identify and measure two main dimensions in the emotional structure: Positive Affect (PA) and Negative Affect (NA) (Watson and Clark, 1994). Widely applied in the field of psychology, it is an authoritative scale used to measure emotional states within a specific time frame as well as personality traits.

The PANAS-X contains 55 words describing specific emotions, which are divided into 11 dimensions: Fear, Hostility, Guilt, Sadness, Joviality, Self-Assurance, Attentiveness, Shyness, Fatigue, Serenity, and Surprise. The first four dimensions belong to the Negative Affect part, the middle three to the Positive Affect part, and the last four to the “other affect” part. The PANAS-X adopts a 5-point Likert scale, with 1 rated as “Very slightly or not at all” and 5 as “Extremely.”

The PANAS-X (Chinese Version), revised and validated by Guo and Gan, is an adaptation based on the Chinese cultural context (Guo and Gan, 2010). Compared with the original PANAS-X, the Chinese version has removed the two dimensions of “Fear” and “Attentiveness.” Its reliability and validity have been verified, and the retest results are favorable. The Cronbach’s α coefficient of the total scale is 0.976, and the α coefficients of each subscale range from 0.639 to 0.905. The retest reliability is 0.69 after 1 week and 0.64 after 1 month. Confirmatory factor analysis shows that the scale has good construct validity (*χ*^2^/df = 2.60, RMSEA = 0.07, CFI = 0.96), making it suitable for research on the Chinese population (Guo and Gan, 2010). In this study, the PANAS-X (Chinese Version) was used to measure the emotional states of the subjects within 1 week.

In this study, the nine emotional dimensions of the Chinese version of the PANAS-X were analyzed as continuous variables. Each dimension’s score ranged from 1 to 5 (with 3–8 items per dimension rated on a 5-point Likert scale). To visually illustrate the distributional differences and explore potential associations between emotional states and PAMSS, we conducted an exploratory subgrouping based on sample percentiles:

- High emotion group: Scores ≥ 75^th^ percentile of the sample distribution.
- Low emotion group: Scores ≤ 25^th^ percentile of the sample distribution.

Example: For the hostility dimension [scores ranged: 1–5], the 75th percentile was 4; thus, scores ≥4 defined the high hostility group.

This grouping strategy was based on the sample’s internal distribution rather than any predefined theoretical thresholds. It aimed to capture individuals at the distributional extremes of each emotional dimension and to preliminarily identify potential association patterns. Such percentile-based subgrouping is commonly used in exploratory affective science research to examine trends within natural variability (Watson and Clark, 1999; Huang and Zhang, 2003; Kuppens et al., 2010).

Nevertheless, we acknowledge the methodological limitations of dichotomizing continuous variables, such as reduced statistical power and loss of nuanced information. Accordingly, the subgroup analysis was conducted solely for exploratory and illustrative purposes, not as a basis for explanatory conclusions. All findings involving emotional subgroup differences should be interpreted as hypothesis-generating and viewed with appropriate caution.

### 2.6 Statistical analysis

All analyses were conducted using SPSSAU software,^2^ with statistical significance defined as *p* < 0.05. Effect sizes were reported for all inferential tests. The analytical framework consisted of the following two parts:

#### 2.6.1 Descriptive statistics and between-group comparisons

- Descriptive statistics: The prevalence rates of musculoskeletal symptoms were described using frequencies and percentages (%). Maximum, minimum, and mean values quantified the scores of pain/discomfort across body regions.
- Chi-square tests: Cross-tabulation analyses were used to examine distribution differences of symptoms across playing habit categories. Metrics: *χ*^2^, *p*-value.
- Independent samples t-tests: Differences in emotional scores (PANAS-X dimensions) between symptomatic and asymptomatic groups were compared. Metrics reported included *t* values, Cohen’s *d*, *p* values, and mean differences with 95% confidence intervals.
- Analysis of variance (ANOVA): Differences in the maximum, minimum, and mean pain or discomfort scores were analyzed across different grade levels and playing postures.

#### 2.6.2 Correlation and regression modeling

- Spearman rank correlation (*ρ*): Evaluated associations between symptom status (binary: 0 = asymptomatic, 1 = symptomatic) and emotion scores. Metrics: *ρ*, *p* value.
- Pearson correlation analysis: Examined the relationships between pain or discomfort intensity in each body region and PAMSS, as well as emotional states. Indicators included correlation coefficient (*r*) and significance level (*p*).
- Exploratory stepwise linear regression: Stepwise regression was employed to explore associations between pain or discomfort intensity in each body region and PAMSS. The entry criterion was set at *p* < 0.05 and the removal criterion at *p* > 0.10. Multicollinearity was assessed using variance inflation factors (VIF < 5). Model fit was evaluated using adjusted *R*^2^.
- Binary logistic regression: A backward stepwise binary logistic regression model was constructed to analyze associated factors of PAMSS in different body regions, including demographic characteristics, playing habits, and playing postures. Model evaluation was conducted using Nagelkerke *R*^2^.

## 3 Results

### 3.1 Descriptive statistical analysis

Descriptive statistical analysis results showed that 53 out of 106 samples tested positive for PAMSS, accounting for 50% of the total samples. For the PAMSS group, the prevalence rates of PAMSS in different body parts, listed from high to low, were as follows: Neck (25.47%), Shoulder (23.58%), Wrists/Hands (22.64%), Lower Back (22.64%), Upper Back (17.92%), Forearms (12.26%), Upper Arms (11.32%), Hips/Thighs (8.49%), Elbows (6.60%), Calf (5.66%), Knees (3.77%), and Ankles/Feet (3.77%). Notably, the five body parts with the highest prevalence of PAMSS were Neck, Shoulder, Wrists/Hands, Lower Back, and Upper Back, with an obvious concentration in the upper limbs (Table 1).

**Table 1:** Overall description (PAMSS in different body regions).

Frequency analysis results				
Category	Option	Frequency	Percentage (%)	Cumulative percentage (%)
PAMSS group	No	53	50	100
	Yes	53	50	50
Ankles/feet	0	102	96.23	96.23
	1	4	3.77	100
Calf	0	100	94.34	94.34
	1	6	5.66	100
Knees	0	102	96.23	96.23
	1	4	3.77	100
Hips/thighs	0	97	91.51	91.51
	1	9	8.49	100
Wrists/hands	0	82	77.36	77.36
	1	24	22.64	100
Lower back	0	82	77.36	77.36
	1	24	22.64	100
Forearms	0	93	87.74	87.74
	1	13	12.26	100
Elbows	0	99	93.4	93.4
	1	7	6.6	100
Upper arms	0	94	88.68	88.68
	1	12	11.32	100
Upper back	0	87	82.08	82.08
	1	19	17.92	100
Shoulder	0	81	76.42	76.42
	1	25	23.58	100
Neck	0	79	74.53	74.53
	1	27	25.47	100
Total		106	100	100

0 = No symptoms, 1 = With piano-associated musculoskeletal symptoms (PAMSS).

The chi-square test indicated significant differences in overall positive cases in terms of gender and student status, but no significant differences were detected in relation to grade level (*p* = 0.686 > 0.05). Specifically, gender showed significant differences at the 0.01 level (*χ*^2^ = 6.989, *p* = 0.008 < 0.01). A comparison of percentages revealed that 57.69% of female participants reported having disorders, significantly higher than the 28.57% of males. Regarding student status, significant differences were observed at the 0.05 level (*χ*^2^ = 6.697, *p* = 0.035 < 0.05). Among university students and music conservatory students, 56.10 and 55.32% reported having disorders, respectively, both higher than the average of 50%.

Moreover, the chi-square test revealed that gender had an impact on differences in affected body parts among positive cases. Specifically, the incidence of neck disorders showed a significant difference (*χ*^2^ = 6.734, *p* = 0.009 < 0.01). No significant differences were found between student status, grade level, and the distribution of affected body parts among positive cases. A percentage comparison showed that 32.05% of females reported neck disorders, significantly higher than the 7.14% of males, suggesting that females might be more prone to neck disorders than males.

Firstly, descriptive statistical analysis results show that, according to the 0–10 scale for pain or discomfort levels in different body parts, the minimum pain or discomfort level in all body parts was 0. The maximum pain or discomfort levels were 10 for the Neck, Shoulder, Upper Back, Lower Back, and Wrists; 8 for the Upper Arms, Elbows, Forearms, Hips/Thighs, and Knees; 7 for Ankles/Feet; and 6 for the Calf. The average pain or discomfort levels for different body parts, ranked from highest to lowest, were as follows: Neck (3.07), Shoulder (3.04), Lower Back (2.60), Wrists/Hands (2.56), Upper Back (1.82), Forearms (1.37), Upper Arms (1.09), Knees (0.88), Hips/Thighs (0.81), Elbows (0.76), Ankles/Feet (0.60), and Calf (0.60). The five body parts with the highest pain or discomfort levels were Neck, Shoulder, Lower Back, Wrists/Hands, and Upper Back, primarily concentrated in the upper limbs, consistent with the areas of high prevalence.

Secondly, T-test analysis found a significant difference in the pain or discomfort levels for the Neck based on gender at the 0.01 significance level (*t* = −2.837, *p* = 0.005 < 0.01). More precisely, the average for males (1.82) was significantly lower than for females (3.51) (Table 2). Lastly, ANOVA analysis of the differences in pain or discomfort levels based on student status and grade revealed no significant difference in pain or discomfort levels based on student status. Nevertheless, grade level showed significant differences at the 0.05 level for the Upper Arms and Lower Back (Waist), with specific findings as follows: Upper Arms (*F* = 2.859, *p* = 0.013), with the following group average comparisons: Sophomore > Freshman; Sophomore > Junior; Sophomore > Senior; Sophomore > Third-year Graduate; Second-year Graduate > Senior. Lower Back (Waist) (*F* = 2.491, *p* = 0.028), with the following group average comparisons: Sophomore > Freshman; Second-year Graduate > Freshman; Sophomore > Senior; Second-year Graduate > Junior; Second-year Graduate > Senior; Second-year Graduate > First-year Graduate.

**Table 2:** Descriptive statistics of pain or discomfort intensity by body regions and gender in PAMSS.

Name	Minimum	Maximum	M (SD)	Median	Males M (SD)	Females M (SD)
Neck**	0	10	3.066 (2.795)	3.000	1.82 (2.37)	3.51 (2.81)
Shoulder	0	10	3.038 (2.982)	3.000	2.21 (2.79)	3.33 (3.01)
Upper back	0	10	1.821 (2.589)	0.000	1.43 (2.39)	1.96 (2.66)
Upper arms	0	8	1.085 (1.888)	0.000	0.86 (1.63)	1.17 (1.98)
Elbows	0	8	0.755 (1.667)	0.000	0.96 (2.13)	0.68 (1.47)
Forearms	0	8	1.368 (2.072)	0.000	1.21 (2.22)	1.42 (2.03)
Lower back	0	10	2.604 (3.182)	1.000	1.93 (2.85)	2.85 (3.28)
Wrists/hands	0	10	2.557 (2.839)	2.000	2.68 (3.04)	2.51 (2.78)
Hips/thighs	0	8	0.811 (1.628)	0.000	0.75 (1.60)	0.83 (1.65)
Knees	0	8	0.877 (1.896)	0.000	0.86 (1.72)	0.88 (1.97)
Calf	0	6	0.594 (1.286)	0.000	0.82 (1.61)	0.51 (1.15)
Ankles/feet	0	7	0.594 (1.379)	0.000	0.71 (1.44)	0.55 (1.36)

Male (*N* = 28); Female (*N* = 78). *p < 0.05. **p < 0.01.

### 3.2 Differential analysis

Relationship between Associated Factors and PAMSS Impact.

Chi-square tests were also used to examine distribution differences of musculoskeletal disorders across playing habits. No significant differences were found for natural relaxed breathing, breathing awareness, warm-up habits, sitting postures, and musculoskeletal disorders in the different body parts (*p* > 0.05).

Certain aspects of playing habits were significantly associated with disorders in specific body parts:

- Relaxation state after key press: Hips/Thighs (*χ*^2^ = 7.786, *p* = 0.020 < 0.05), Upper Back (*χ*^2^ = 7.720, *p* = 0.021 < 0.05), Neck (*χ*^2^ = 14.393, *p* = 0.001 < 0.01).
- Shoulder abduction posture: Hips/Thighs (*χ*^2^ = 6.611, *p* = 0.010 < 0.05), Lower Back (*χ*^2^ = 8.053, *p* = 0.005 < 0.01), Forearms (*χ*^2^ = 6.313, *p* = 0.012 < 0.05), Upper Arms (*χ*^2^ = 7.455, *p* = 0.006 < 0.01), Upper Back (*χ*^2^ = 4.908, *p* = 0.027 < 0.05), Shoulders (*χ*^2^ = 4.252, *p* = 0.039 < 0.05).
- Wrist posture: Hips/Thighs (*χ*^2^ = 5.894, *p* = 0.015 < 0.05), Elbows (*χ*^2^ = 4.004, *p* = 0.045 < 0.05).
- Shoulder shrugging: Shoulders (*χ*^2^ = 6.675, *p* = 0.036 < 0.05), Neck (*χ*^2^ = 9.956, *p* = 0.007 < 0.01).
- Seat height setting: Knees (*χ*^2^ = 4.467, *p* = 0.035 < 0.05), Forearms (*χ*^2^ = 4.765, *p* = 0.029 < 0.05).

### 3.3 Exploratory correlations analysis

#### 3.3.1 The association between pain or discomfort intensity and PAMSS in different body regions (Table 3)

**Table 3:** Association between pain or discomfort intensity and PAMSS in different body regions.

Body region	Correlation (*r*)	*p* value
Upper arms	0.349	<0.01
Neck	0.342	<0.01
Upper back	0.300	<0.01
Elbows	0.287	<0.01
Hips/thighs	0.257	<0.01
Forearms	0.241	<0.05
Calf	0.232	<0.05
Wrists/hands, knees, ankles/feet, lower back, shoulder	Not significant	>0.05

Correlation analysis revealed that pain/discomfort intensity in the Upper Arms, Neck, Upper Back, Elbows, Hips/Thighs, Forearms, and Calf was significantly and positively associated with the presence of PAMSS in the corresponding regions. The strongest associations were in the Upper Arms (*r* = 0.349), Neck (*r* = 0.342), and Upper Back (*r* = 0.300).

#### 3.3.2 The association between pain or discomfort intensity and emotional states in different body regions (Table 4)

**Table 4:** Association between pain or discomfort intensity and emotional states in different body regions.

Body region	Emotional states	Correlation (*r*)	*p* value
Wrists/hands	Surprise	±0.357	<0.01
Elbows	Surprise	±0.324	<0.01
Hips/thighs	Surprise	±0.301	<0.01
Forearms	Surprise	±0.294	<0.01
Upper back	Surprise	±0.281	<0.01
Calf	Surprise	±0.224	<0.05
Knees	Surprise	±0.194	<0.05
Neck	Fatigue	0.231	<0.05

Further analysis showed that surprise was bidirectionally associated with pain/discomfort intensity in several regions (*r* = −0.194 to +0.357, *p* < 0.05), with stronger associations in Wrists/Hands (*r* = ±0.357) and Elbows (*r* = ±0.324). Fatigue was positively correlated with Neck pain/discomfort intensity (*r* = 0.231, *p* < 0.05). The original 0–10 scale was dichotomized to explore associations between pain/discomfort intensity and PAMSS. Scores 0–3 were coded as “mild or no discomfort” (0), and scores ≥4 as “moderate to severe discomfort” (1). This cutoff follows CDC guidance, which supports using pain scales for screening and monitoring rather than diagnosis (Dowell et al., 2022).

### 3.4 Stepwise linear regression analyses

To further explore whether moderate-to-severe pain intensity in specific body regions is statistically associated with PAMSS, stepwise linear regression models were performed for regions without evident multicollinearity. The final models identified several body regions in which pain intensity was significantly associated with PAMSS. All included variables passed multicollinearity and independence diagnostics. Other body regions were not included in the final regression models due to multicollinearity or insufficient statistical power. Detailed results are presented in Table 5.

**Table 5:** Association between moderate-to-severe pain intensity and PAMSS across body regions.

Body region	Pain variable included	*β* (Standardized)	95% CI	*p*-value	Adjusted *R*^2^	*F*-statistic	D–W statistic
Neck	Moderate/severe pain in neck	0.303	0.143–0.462	<0.01	0.109	*F* (1,104) = 13.814, *p* < 0.001	1.999
Shoulder	Moderate/severe pain in shoulder	0.313	0.160–0.466	<0.01	0.125	*F* (1,104) = 16.032, *p* < 0.001	1.669
Forearm	Moderate/severe pain in forearm	0.238	0.044–0.431	<0.05	0.044	*F* (1,104) = 5.782, *p* = 0.018	2.072
Wrists/hands	Moderate/severe pain in wrists/hands	0.227	0.056–0.398	<0.05	0.052	*F* (1,104) = 6.748, *p* = 0.011	2.045

### 3.5 Binary logistic regression analysis for PAMSS

Binary logistic regression models were constructed using the backward selection method to analyze associations between PAMSS across different body regions and demographic characteristics, playing habits, and playing postures. Model performance was evaluated using Nagelkerke’s pseudo R-squared (Nagelkerke *R*^2^). Key findings from the regression analyses are summarized in Table 6, with salient results detailed below.

**Table 6:** Multivariable logistic regression analysis of PAMSS by body regions^a^.

Body region	Nagelkerke *R*^2^	Predictor (reference group)^c^	OR (95% CI)^b^	*p*-value	Effect direction^d^
Neck	0.253	Female (vs Male)	6.28 (1.28–30.86)	0.024	↑
		Barely relaxing after key touch (vs Usually relaxing)	4.73 (1.29–17.36)	0.019	↑
Shoulder	0.242	Female (vs Male)	4.24 (1.03–17.44)	0.045	↑
		Barely notice breathing (vs Regular breathing)	3.30 (1.20–9.09)	0.021	↑
		Unnatural shoulder abduction (vs Natural)	4.33 (1.14–16.51)	0.032	↑
Upper back	0.130	Barely relaxing after key touch (vs Usually relaxing)	3.97 (1.17–13.51)	0.027	↑
		Unnatural shoulder abduction (vs Natural)	3.52 (1.05–11.84)	0.042	↑
Lower back	0.243	Female (vs Male)	4.18 (1.06–16.54)	0.041	↑
		Mild kyphotic sitting (vs Vertical sitting)	3.70 (1.13–12.16)	0.031	↑
		Unnatural shoulder abduction (vs Natural)	6.97 (1.85–26.29)	0.004	↑
		Unnatural chair height (vs Natural)	0.25 (0.07–0.97)	0.045	↓
*↑ Subtypes-Chair Height Settings* ^e^	0.250	Elbow parallel keyboard (vs Below keyboard)	4.88 (1.09–21.81)	0.038	↑
	0.250	Elbow above keyboard (vs Below keyboard)	0.21 (0.05–0.92)	0.038	↓
Upper Arms	0.157	Unnatural shoulder abduction (vs Natural)	5.36 (1.40–20.64)	0.015	↑
Elbows	0.201	Barely relaxing after key touch (vs Usually relaxing)	6.87 (1.10–42.80)	0.039	↑
Forearms	0.151	Unnatural shoulder abduction (vs Natural)	4.38 (1.16–16.45)	0.029	↑
Hips/Thighs	0.208	Barely relaxing after key touch (vs Usually relaxing)	6.44 (1.37–30.15)	0.018	↑
		Unnatural shoulder abduction (vs Natural)	5.84 (1.25–27.23)	0.025	↑
Knees	0.287	Unnatural wrist posture (vs Natural)	20.22 (1.29–315.93)	0.032	↑
		Age (per year)	1.48 (1.08–2.04)	0.016	↑

^a^Includes only variables with *p* < 0.10.

^b^OR: odds ratio.

^c^Reference category follows “vs”.

^d^Effect direction: ↑ = positive association (OR > 1), ↓ = negative association (OR < 1).

^e^*↑Subtypes-Chair Height Settings*: Detailed analysis of chair height predictors for lower back symptoms.

#### 3.5.1 Summary of key associations

Overall, several predictors demonstrated significant associations with symptoms across multiple body regions. Female gender showed significant positive associations with symptoms in the neck, shoulder, and lower back regions (OR range: 4.18–6.28). Unnatural shoulder abduction postures were significantly associated with symptoms in the shoulders, upper back, lower back, upper arms, forearms, and hips/thighs (OR range: 3.52–6.97). Insufficient key-touch relaxation exhibited significant positive associations with symptoms in the neck, upper back, elbows, and hips/thighs (OR range: 3.97–6.87).

#### 3.5.2 Region-specific findings

- Neck symptoms: Significant associations were found with female gender (OR = 6.28) and insufficient key-touch relaxation (OR = 4.73).
- Shoulder symptoms: Female gender (OR = 4.24), unnatural shoulder abduction posture (OR = 4.33), and lower breathing awareness (OR = 3.30) were significantly associated with symptoms.
- Upper back symptoms: Significant associations were observed with insufficient key-touch relaxation (OR = 3.97) and unnatural shoulder abduction (OR = 3.52). The model had relatively low explanatory power (*R*^2^ = 0.130).
- Lower back symptoms: Significant positive associations were found with female gender (OR = 4.18), mild kyphotic sitting (OR = 3.70), and unnatural shoulder abduction (OR = 6.97), while chair height setting was significantly negatively associated (OR = 0.25). A subtype analysis (see Table 3, footnote ↑) revealed that, compared to standard posture: - Elbows parallel to the keyboard were associated with increased symptom likelihood (OR = 4.88). - Elbows above the keyboard were associated with decreased symptom likelihood (OR = 0.21).
- Upper arms symptoms: Unnatural shoulder abduction was significantly associated (OR = 5.36).
- Elbows symptoms: A significant association was observed with insufficient key-touch relaxation (OR = 6.87).
- Forearms symptoms: Unnatural shoulder abduction was significantly associated (OR = 4.38).
- Hips/Thighs symptoms: Significant associations were found with both insufficient key-touch relaxation (OR = 6.44) and unnatural shoulder abduction (OR = 5.84).
- Knees symptoms: Significant associations were identified for abnormal wrist posture (OR = 20.22; 95% CI: 1.29–315.93), indicating the need for cautious interpretation. Age was also significantly associated (OR = 1.48 per year increase; 95% CI: 1.08–2.04, *p* = 0.016). This model showed the highest explanatory power (*R*^2^ = 0.287).
- Ankles/feet symptoms: No statistically significant predictors were identified.

#### 3.5.3 Model explanatory power

Substantial variation in Nagelkerke *R*^2^ values across regions (range: 0.130–0.287) was observed. Limited explanatory power in the upper back model (*R*^2^ = 0.130) suggests unaccounted confounding variables.

### 3.6 Between-group differences in emotions states based on PAMSS

In the analysis of the relationship between emotions and musculoskeletal symptoms, we found that the data for upper limb regions (upper arms, shoulder, and upper back) exhibited the strongest and most consistent correlations with emotional states. Therefore, to streamline the analysis and focus on the most relevant findings, we chose to analyze only the symptoms in these three upper limb regions. Results for other body parts, which showed weaker or no significant relationships with emotional states, were excluded from further analysis.

To explore the relationship between PAMSS and emotional states, independent samples t-tests were conducted to compare emotional scores (measured by PANAS-X dimensions) between participants with and without symptoms in specific upper limb regions. The analysis aimed to determine whether musculoskeletal symptoms were associated with significant differences in emotional experiences.

The results revealed that participants with symptoms in the upper arms, shoulders, and upper back reported significantly higher scores in certain negative emotional states compared to their asymptomatic counterparts (see Table 7):

**Table 7:** Comparison of emotional states in relation to musculoskeletal symptoms in the upper arms, shoulder, and upper back.

Emotion	Body region	*t*	*p*	Cohen’s *d*
Surprise	Upper arms	−3.802	0.000**	−0.745
	Shoulder	−2.155	0.033*	−0.422
	Upper back	−3.209	0.002**	−0.629
Sadness	Upper arms	−1.936	0.056	−0.38
	Shoulder	−1.2	0.233	−0.235
	Upper back	−2.491	0.014*	−0.488
Self-assurance	Upper arms	−1.829	0.07	−0.358
	Shoulder	0.463	0.645	0.091
	Upper back	−0.26	0.795	−0.051
Serenity	Upper arms	0.727	0.469	0.143
	Shoulder	0.271	0.787	0.053
	Upper back	1.024	0.308	0.201
Guilt	Upper arms	−3.717	0.000**	−0.728
	Shoulder	−2.556	0.015*	−0.501
	Upper back	−3.012	0.003**	−0.59
Shyness	Upper arms	−2.135	0.035*	−0.418
	Shoulder	−1.687	0.095	−0.331
	Upper back	−1.354	0.179	−0.265
Fatigue	Upper arms	−1.12	0.265	−0.22
	Shoulder	−1.555	0.123	−0.305
	Upper back	−1.333	0.185	−0.261
Joviality	Upper arms	−1.585	0.116	−0.311
	Shoulder	0.013	0.99	0.003
	Upper back	0.72	0.473	0.141
Hostility	Upper arms	−2.014	0.047*	−0.395
	Shoulder	−1.196	0.235	−0.234
	Upper back	−2.066	0.051	−0.405

^*^p < 0.05, ^**^p < 0.01.

(a) Upper arm symptom group: Significantly higher scores were observed for Surprise (*t* = −3.802, *d* = −0.745, *p* < 0.001), Guilt (*t* = −3.717, *d* = −0.728, *p* < 0.001), Hostility (*t* = −2.014, *d* = −0.395, *p* = 0.047), and Shyness (*t* = −2.135, *d* = −0.418, *p* = 0.035). Mean differences ranged from 3.8 to 4.6 points (*t* = −2.155, *d* = −0.422, *p* = 0.033) and Guilt (*t* = −2.556, *d* = −0.501, *p* = 0.015), with mean differences ranging from 2.5 to 3.1 points. (b) Upper back symptom group: Significantly higher scores were found in Surprise (*t* = −3.209, *d* = −0.629, *p* = 0.002), Sadness (*t* = −2.491, *d* = −0.448, *p* = 0.014), and Guilt (*t* = −3.012, *d* = −0.590, *p* = 0.003), with mean differences ranging from 2.9 to 4.0 points.

These results indicate that musculoskeletal symptoms in the upper limbs (upper arms, shoulders, and upper back) are significantly associated with negative emotional states including Guilt, Hostility, Sadness, and Surprise. Participants with PAMSS in these regions reported significantly higher emotional scores compared to asymptomatic individuals. All differences showed higher emotional scores in the symptom groups (negative *t* values and negative *d* values in consistent directions).

Surprise and Guilt exhibited stable associations across all affected body regions (all three regions significant, with *d* values ranging from 0.42 to 0.74). The effect sizes were predominantly small to medium (|*d*| = 0.39–0.74), with Surprise showing the largest effect size (maximum *d* = 0.74), followed by Guilt (maximum *d* = 0.72) and Sadness (*d* = 0.45). Hostility and Shyness showed small effect sizes (*d* < 0.42).

### 3.7 Spearman correlation analysis in emotions states based on upper limb PAMSS

To further validate the relationship between upper limb symptoms and emotional dimensions, Spearman’s rank correlation analysis was conducted to examine the association between symptom status (binary: 0 = asymptomatic, 1 = symptomatic) and emotional scores for the upper limb regions (upper arms, shoulder, and upper back) (Table 8). Spearman’s rank correlation was chosen due to the non-parametric nature of the data, as the symptom status variable is binary and no linear or normal distribution of the emotional scores was assumed. The results revealed the following significant positive correlations: Surprise (*ρ* = 0.32, *p* < 0.001), Guilt (*ρ* = 0.29, *p* = 0.002), Shyness (*ρ* = 0.21, *p* = 0.031), and Hostility (*ρ* = 0.19, *p* = 0.049).: Surprise (*ρ* = 0.22, *p* = 0.024) and Guilt (*ρ* = 0.23, *p* = 0.018).: Surprise (*ρ* = 0.30, *p* = 0.001), Sadness (*ρ* = 0.24, *p* = 0.012), and Guilt (*ρ* = 0.27, *p* = 0.004).

**Table 8:** Spearman’s rank correlation between upper limb musculoskeletal symptoms and emotions states.

Body region	Emotion	*ρ* Value	*p* Value
Upper arms	Surprise	0.32	<0.001
Upper arms	Guilt	0.29	0.002
Upper arms	Shyness	0.21	0.031
Upper arms	Hostility	0.19	0.049
Shoulder	Surprise	0.22	0.024
Shoulder	Guilt	0.23	0.018
Upper back	Surprise	0.3	0.001
Upper back	Sadness	0.24	0.012
Upper back	Guilt	0.27	0.004

These findings are consistent with the t-test results, further confirming the positive associations between upper limb symptoms and specific emotional states, particularly Surprise and Guilt.

The symptom variable was binary, and the Spearman correlations reflect relationship between this binary variable (symptom status) and continuous emotional scores. Although the correlations were statistically significant, the coefficients (*ρ*) were relatively small (all < 0.32). This suggests that while the observed associations are significant, their clinical significance may be limited, potentially influenced by the binary nature of symptom coding, sample size, and unexamined confounding variables.

### 3.8 Emotions states grouping and its relationship with upper limb PAMSS

To further explore the relationship between emotional states and upper limb musculoskeletal symptoms, participants were grouped into high and low emotional states based on the 75th and 25th percentiles of each emotional dimension. For example, a score ≥ 3 on Surprise was classified as high Surprise, while a score ≤ 1 was classified as low Surprise.

The results indicated that participants in the high emotion groups exhibited significantly higher rates of PAMSS in the upper arms, shoulder, and upper back regions compared to those in the low emotion groups (Table 9). This trend was particularly evident for negative emotions, including Hostility, Sadness, and Guilt, as well as for Surprise, where the symptom index (SI) values were consistently elevated in the high emotion groups (e.g., Guilt–Upper Arms: 40.00% vs. 23.81%).

**Table 9:** Associated factors of playing musculoskeletal symptoms across emotions states groups.

Emotion	Emotion group	75th percentile	25th percentile	Upper arms SI	Shoulder SI	Upper back SI
Hostility	High	3	1	33.33%	33.33%	36.11%
	Low	1	1	19.51%	24.39%	21.95%
Sadness	High	3	1	44.12%	47.06%	41.18%
	Low	1	1	21.74%	21.74%	17.39%
Surprise	High	3	1	39.39%	36.36%	36.36%
	Low	1	1	24.44%	20.00%	22.22%
Joviality	High	4	2	22.73%	22.73%	20.45%
	Low	2	2	25.93%	25.93%	22.22%
Self-assurance	High	4	2	20.00%	20.00%	20.00%
	Low	2	1	32.14%	32.14%	28.57%
Shyness	High	3	1	56.25%	53.13%	50.00%
	Low	1	1	14.29%	12.24%	12.24%
Fatigue	High	4	2	23.08%	23.08%	20.51%
	Low	2	2	19.23%	23.08%	19.23%
Guilt	High	3	1	40.00%	40.00%	34.29%
	Low	1	1	23.81%	21.43%	21.43%
Serenity	High	4	2	13.95%	13.95%	11.63%
	Low	2	2	16.00%	16.00%	12.00%

SI, Symptoms incidence.

Among the emotions analyzed, Shyness showed the highest PAMSS index in the upper arms, with the high emotion group reporting an SI of 56.25% compared to 14.29% in the low emotion group. Similarly, the high emotion group for Guilt had an SI of 40.00% in the upper arm and 34.29% in the upper back, while the low emotion group had values of 23.81 and 21.43%, respectively.

Hostility and sadness also exhibited high SI values in the upper arms and shoulder regions, with the high emotion groups reporting 33.33 and 44.12% respectively, compared to the low emotion groups with 19.51% for Hostility and 21.74% for Sadness. Surprise also showed a notable difference in the shoulder region, with the high emotion group experiencing an SI of 36.36%, compared to 20.00% in the low emotion group.

These findings were consistent with the results from independent samples t-tests and Spearman correlation analyses, showing a significant positive correlation between higher emotional intensity (especially in negative emotions like Guilt, Hostility, Sadness, and Surprise) and upper limb PAMSS.

## 4 Discussion

In China, the prevalence of musculoskeletal symptoms among Chinese college students who play piano is 50%, higher than the prevalence of PAMSS reported among Malaysian college students in higher education institutions (34.5%) (Ling et al., 2018) and the prevalence reported by Furuya et al. (41%) (Furuya et al., 2006). The high-incidence body parts among Chinese college piano students are the neck (25.47%), shoulder (23.58%), wrists/hands (22.64%), lower back (22.64%), and upper back (17.92%), predominantly concentrated in the upper limbs, which aligns with findings from other studies (Li et al., 2024; Zhao et al., 2024).

This study also explored the association between self-reported discomfort and PAMSS. Based on stepwise regression analysis, moderate to severe discomfort (NRS ≥ 4) in four upper-limb regions was significantly associated with the presence of PAMSS in the corresponding body parts (*p* < 0.05): neck (*β* = 0.31, 95% CI: 0.12–0.50), shoulders (*β* = 0.28, 95% CI: 0.09–0.47), forearms (*β* = 0.25, 95% CI: 0.07–0.43), and wrists/hands (*β* = 0.23, 95% CI: 0.05–0.41). This proximal-to-distal gradient of association may reflect the load transmission patterns along the upper-limb kinetic chain during piano performance.

Several studies have discussed possible mechanisms underlying this trend. Singh et al. reported that chair-related factors, such as seat height, tilt, and back support, may affect upper-limb musculoskeletal load during precision tasks (Singh et al., 2016). Furthermore, Turner et al. found that pianists’ proximal motor strategies influence upper-limb movement variability and load distribution; insufficient control of the shoulder and elbow may result in compensatory overuse of the wrist and hand (Turner et al., 2023). These findings suggest that the occurrence of upper-limb PAMSS may be associated with both playing technique and external postural support conditions.

While discomfort intensity may be influenced by individual playing habits or postures, in this study we primarily employed it as a self-reported perceptual metric to identify potential musculoskeletal risks. Consequently, we did not model it as a primary outcome variable. The observed effect sizes suggest that regional discomfort scores could function as a preliminary screening tool in clinical or educational settings. Future studies should incorporate longitudinal designs, larger samples, and penalized regression methods (e.g., ridge regression) to enhance model robustness and elucidate the potential etiological role of discomfort intensity in PAMSS development. Our study further supports these observations by identifying specific posture-associated factors significantly associated with PAMSS, including seat height, wrist postures, shoulder abduction postures, and shoulder shrugging condition. These results are consistent with existing research (Spahn et al., 2002; Allsop and Ackland, 2010; Blanco-Piñeiro et al., 2015; Kok et al., 2016; Li et al., 2024). Therefore, we suggest that performers should maintain a naturally relaxed and dropped shoulder abduction posture, remain in a near-neutral wrist position, and avoid shoulder shrugging during performance to reduce the occurrence of PAMSS.

However, an unexpected finding emerged in this study: the commonly accepted *non-natural* seat height postures (elbows joint above/below the keyboard level) were negatively correlated with lower back PAMSS, indicating a lower incidence of symptoms. This finding contradicts conventional ergonomic viewpoints. Drawing on ergonomic literature, we hypothesize that compensatory postural adjustments resulting from mismatched piano and seat heights, changes in muscle fatigue distribution due to prolonged static loading, and individual differences in body proportions (anthropometric variations) may contribute to the occurrence of PAMSS (Gates and Dingwell, 2011; Singh et al., 2016; Ohlendorf et al., 2017; Filiz Ozdemir et al., 2021; Irfan et al., 2023; Turner et al., 2023). Notably, exploration of these factors in the context of PAMSS or PRMDs remains relatively limited, possibly because existing research has predominantly focused on other aspects. Therefore, future studies should expand and complement the current body of knowledge from an ergonomic perspective, investigating how these factors influence performers’ postures and muscle load, ultimately contributing to musculoskeletal symptom development.

Moreover, we found that certain playing postures may not only affect the corresponding body parts but may also impact more distant areas. However, the limitations of the self-report questionnaire prevent us from uncovering the mechanisms underlying this phenomenon. Future investigations could employ technologies such as motion capture and electromyography (EMG) for more in-depth analysis.

This study examined the impact of playing habits on PAMSS, revealing that excessive muscle tension caused by continuous force exertion after key touch and failure to relax increases the risk of disorders in the shoulders, neck, upper back, elbows, and hips/thighs. It also demonstrated that a lack of timely relaxation during key touch may increase neuromuscular load and contribute to the development of chronic upper limb injuries. This finding partially supports the longitudinal study by Cruder et al. and validates the subjective experiences and theories on playing habits proposed by Mark, Hamilton, and others in pedagogical literature (Hamilton, 2012; Mark et al., 2003; Cruder et al., 2023), confirming that the relaxation state after key touch and breathing awareness influence musculoskeletal symptoms. Further analysis revealed that this habit does not significantly affect proximal structures, such as the forearms and upper arms, but more frequently affects the body’s core support regions. This suggests that long-chain muscle groups, when under high tension, may compensate by transferring the load to the trunk and stabilizing regions such as the shoulders, neck, upper back, and pelvic area (Neumann, 2010). Therefore, if performers fail to release tension after exertion, the muscles may remain in prolonged isometric contraction, increasing the load on deep stabilizing muscles and raising the risk of injury to distal body parts. A systematic review by Overton et al. highlighted that musicians with musculoskeletal symptoms often experience increased neuromuscular load, which further supports this hypothesis (Overton et al., 2018).

Regarding breathing, a lack of breathing awareness during piano playing may increase musculoskeletal symptoms of the shoulder. We speculate that this is closely related to the principle of breathing. On one hand, breathing is a passive and unconscious process in a calm state. However, passive breathing alone cannot provide sufficient oxygen required by the brain and muscles during piano playing. Therefore, a greater intensity of breathing is required to support the body’s energy supply. Lack of oxygen may cause muscle fatigue, thus triggering disorders (Neumann, 2010, p. 74). On the other hand, active breathing engages shoulder muscles like the serratus anterior and levator scapulae, aiding chest expansion and inhalation. During exhalation, these muscles relax, maintaining the shoulders in a relaxed, elastic state. Such conscious breathing continuously mobilizes the shoulder muscles to contract and relax, keeping them in a relaxed and elastic state. Therefore, when there is a lack of active breathing awareness, the shoulders may remain stationary and stiff for long periods, leading to disorders (Neumann, 2010, pp. 454–61). Furthermore, Stanhope suggested that breathing disorders may induce muscle tension through sympathetic nervous system activation, highlighting their potential link to musculoskeletal disorders (Stanhope et al., 2022). Therefore, a lack of breathing awareness may affect the occurrence of shoulder PAMSS through multiple interacting pathways.

This study found that gender differences play a significant role in the occurrence of PAMSS. More female students reported PAMSS compared to male students, which is consistent with previous research (Kok et al., 2016; Li et al., 2024). In terms of subjective perception, female participants reported significantly higher pain intensity levels in the neck, upper back, shoulder, lower back, hips/thighs, and wrists/hands compared to male participants. This gender difference may be influenced by multiple factors, including generally smaller hand size in females, hormonal differences, and societal and cultural influences on pain expression (Boyle, 2013; De la Corte-Rodriguez et al., 2024). Moreover, psychological and emotional responses to pain, which often differ by gender, may also play a significant role (Kenny and Ackermann, 2014). Understanding these underlying factors could provide a more comprehensive view of the gender differences observed in the prevalence of PAMSS.

This study also found a significant association between negative emotions, including hostility, guilt, sadness, and surprise, and the occurrence of upper limb PAMSS in Chinese piano students. Specifically, participants with symptoms in the upper arm, shoulder, and upper back reported significantly higher scores on emotional dimensions such as surprise, guilt, hostility, and sadness compared to those without symptoms, with all effects showing consistent directionality (i.e., negative *t* and *d* values). Among these, surprise (maximum Cohen’s *d* = 0.745) and guilt (maximum *d* = 0.728) demonstrated moderate effect sizes across all three regions, suggesting relatively stable cross-site associations. Sadness showed a small-to-moderate effect (*d* = 0.448), while hostility and shyness were associated with small effects (*d* < 0.42). Although most effect sizes ranged from small to moderate, the differences in emotional scores (particularly in the upper arm and upper back groups) reached relatively stronger magnitudes, implying potential practical significance.

These exploratory findings align with the multidimensional interaction framework proposed by the biopsychosocial model (Gatchel et al., 2007; Guptill, 2008), which suggests that physical symptoms may be shaped by the interplay of emotional states, psychological responses, and behavioral or social factors. The observed emotional differences in this study point to potential associations between specific emotional profiles and symptom presentation, supporting a multifactorial perspective on understanding pianists’ physical discomfort experiences.

According to Kenny et al., emotional states can influence spinal cord excitability through the limbic system, which in turn affects muscle tension and pain perception (Kenny and Ackermann, 2014; Kenny et al., 2016). Emotional suppression and performance anxiety are likely to lead to sustained muscle tension, potentially exacerbating symptoms. In the behavioral feedback loop, the onset of PAMSS can restrict performance, triggering negative emotions such as hostility, which further contribute to a vicious cycle (Miao et al., 2024).

This study found bidirectional correlations between the emotion of *surprise* and pain or discomfort intensity across multiple body regions, suggesting a potentially complex interaction between emotional arousal and localized pain perception. Specifically, wrists/hands and elbows showed moderate positive associations, while knees exhibited a negative correlation, indicating variability in both the direction and strength of associations across anatomical regions. These results suggest the possibility of mutual influence, consistent with earlier findings.

Notably, in this study, surprise emerged as an emotional variable significantly associated with piano-playing musculoskeletal symptoms. Although surprise is typically considered an adaptive and high-arousal emotion, existing research suggests that it often co-occurs with other negatively valenced high-arousal emotions such as fear and anger and may contribute to rapid emotional transitions (Scherer, 2005; Luna and Renninger, 2015; Gu et al., 2019; Neta and Kim, 2023). However, no established theoretical framework currently explains a direct mechanistic link between surprise and musculoskeletal symptoms. The findings in this study should be interpreted as exploratory. They may reflect heightened emotional reactivity to unexpected stimuli in specific performance contexts or operate indirectly through broader stress-associated pathways that influence bodily function. However, these explanations currently lack an established theoretical foundation in performing arts medicine and should therefore be considered as preliminary.

Based on these findings, piano educators may consider exploring the following areas in teaching practices:

- encouraging students to adopt basic emotional regulation strategies (e.g., cognitive-behavioral techniques) to manage negative emotions associated with musculoskeletal discomfort;
- raising awareness of breathing patterns, as conscious and regular breathing may facilitate muscle relaxation and reduce upper-limb tension;
- reminding students to actively release tension after key touch to prevent excessive muscular force;
- guiding the development of ergonomically informed playing postures, particularly regarding the alignment of the shoulders, neck, elbows, wrists, and fingers;
- considering core and lower-limb stability training to enhance global body support and reduce compensatory upper-limb load.

These pedagogical suggestions are intended as exploratory and should not be interpreted as evidence-based prescriptions. Future longitudinal and interventional studies are needed to validate their effectiveness and inform evidence-based practice in both educational and clinical contexts.

## 5 Conclusion

This study, based on a cross-sectional survey, found that the prevalence of PAMSS among Chinese college piano students is 50%, with symptoms predominantly affecting the neck (25.47%), shoulder (23.58%), wrists/hands (22.64%), lower back (22.64%), and upper back (17.92%). Several posture and playing habit factors were significantly associated with PAMSS, including seat height, non-neutral wrist posture, excessive shoulder abduction, shoulder shrugging, and a lack of breathing awareness and relaxation after key touch. These variables repeatedly appeared in symptoms across multiple body parts, suggesting that they may serve as associated factors. However, this needs further confirmation through longitudinal studies.

Furthermore, PAMSS was significantly associated with several negative emotional dimensions, particularly surprise (*d* = 0.60), guilt (*d* = 0.52), and sadness (*d* = 0.47), which aligns with the biopsychosocial model. This suggests that musculoskeletal symptoms may exacerbate distress through functional impairment, or negative emotions may heighten symptom perception. However, since this study utilized a cross-sectional design, it cannot determine the causal relationship between symptoms and emotions. Future research could validate these findings through longitudinal tracking or experimental designs.

Given that this study used self-reported questionnaires to assess PAMSS and emotional states, there are potential biases related to subjectivity. Moreover, the sample was limited to college piano students, which restricts the generalizability of the findings. In addition, the lack of functional assessment tools (such as the MPIIQM) limits the evaluation of actual performance-related impacts.

Future research should consider the following directions: (a) conduct longitudinal studies to explore the dynamic interplay between the evolution of PAMSS and emotional states; (b) design intervention experiments to test the impact of posture adjustments, relaxation, and breathing training strategies on the incidence of PAMSS; (c) adopt a mixed-methods design, combining quantitative data with qualitative interviews to gain deeper insights into the psychological–behavioral mechanisms underlying PAMSS.

In terms of pedagogical implications, this study suggests greater attention should be given to playing posture, muscle relaxation, and breathing coordination during piano training. Exploring strategies based on existing research on movement rehabilitation and performance anxiety intervention could help maintain performers’ physical health and emotional wellbeing.

## Data Availability

The original contributions presented in the study are included in the article/supplementary material, further inquiries can be directed to the corresponding author.

## Footnotes

^1^ https://www.wjx.cn

^2^ https://spssau.com
